# Manganese Superoxide Dismutase Acetylation and Regulation of Protein Structure in Breast Cancer Biology and Therapy

**DOI:** 10.3390/antiox11040635

**Published:** 2022-03-25

**Authors:** Meredith M. Ogle, Rolando Trevino, Joseph Schell, Mahboubeh Varmazyad, Nobuo Horikoshi, David Gius

**Affiliations:** 1 Department of Radiation Oncology, Mays Cancer Center at UT Health San Antonio MD Anderson, 7979 Wurzbach Road, San Antonio, TX 78229, USA; oglem@uthscsa.edu (M.M.O.); trevinojr@uthscsa.edu (R.T.J.); schellj@uthscsa.edu (J.S.); varmazyad@uthscsa.edu (M.V.); horikoshi@uthscsa.edu (N.H.); 2 Joe R. & Teresa Lozano Long School of Medicine, University of Texas Health San Antonio, 7703 Floyd Curl Drive, San Antonio, TX 78229, USA

**Keywords:** mitochondrial, acetylation, MnSOD, ROS, metabolism, carcinogenesis, redox, sirtuins, electron transport, metal iron metabolism

## Abstract

The loss and/or dysregulation of several cellular and mitochondrial antioxidants’ expression or enzymatic activity, which leads to the aberrant physiological function of these proteins, has been shown to result in oxidative damage to cellular macromolecules. In this regard, it has been surmised that the disruption of mitochondrial networks responsible for maintaining normal metabolism is an established hallmark of cancer and a novel mechanism of therapy resistance. This altered metabolism leads to aberrant accumulation of reactive oxygen species (ROS), which, under specific physiological conditions, leads to a potential tumor-permissive cellular environment. In this regard, it is becoming increasingly clear that the loss or disruption of mitochondrial oxidant scavenging enzymes may be, in specific tumors, either an early event in transformation or exhibit tumor-promoting properties. One example of such an antioxidant enzyme is manganese superoxide dismutase (MnSOD, also referred to as SOD2), which detoxifies superoxide, a ROS that has been shown, when its normal physiological levels are disrupted, to lead to oncogenicity and therapy resistance. Here, we will also discuss how the acetylation of MnSOD leads to a change in detoxification function that leads to a cellular environment permissive for the development of lineage plasticity-like properties that may be one mechanism leading to tumorigenic and therapy-resistant phenotypes.

## 1. Introduction

A fundamental theme in precision, or personalized, cancer medicine is identifying tumors that are vulnerable to specific therapies, based on well-defined molecular biomarkers or tumor signatures [1,2,3,4]. In this regard, estrogen-receptor-positive (ER+) invasive ductal cancers (IDCs), the most common type of breast cancer, are commonly treated with selective estrogen receptor modulators (SERMs), which have been shown in multiple studies to improve clinical outcomes [5,6]. ER+ IDCs are classified as either luminal A or luminal B cancer (LuBCa). LuBCas, which account for most breast cancer deaths in America, exhibit aggressive tumor characteristics, including an elevated proliferative index (high Ki-67); are poorly differentiated (high grade); and display an increased risk of recurrence and metastasis [7,8]. The lethality in women with LuBCa is due, at least in part, to the development of resistance against SERMs and a lack of alternative systemic therapies [9].

The estrogen receptor pathway has a critical role in breast cancer and, therefore, endocrine therapy, which blocks ER signaling, is highly effective; however, over time, a small subset of ER+ tumors recur due to the development of endocrine resistance [7,8]. Multiple mechanisms of endocrine resistance have been identified, including deregulation of various components of the ER signaling pathway, altered cell cycle and cell survival processes, and the activation of escape pathways that provide tumors with alternative proliferative and survival stimuli [5,10]. While most resistance to SERMs involves one of these processes, an increasingly accepted mechanism involves the development of lineage-plasticity-like properties. In this regard, a recent NCI white paper (Beltran, 2019) [11] and a seminal review (Yuan, 2019, *Cancer Discovery*) [12] stated lineage plasticity, due to disruptive microenvironmental cues, stochastic genetics/epigenetics, metabolic alterations, or other therapy-imposed selective pressures, contributes to tumor heterogeneity and, importantly, to the development of resistant phenotypes. 

We define lineage plasticity as a reversible or irreversible reprogramming of cellular systems, where a mature somatic cell can display plasticity, via a change in cell “identity” by dedifferentiation to a progenitor-like state or by transdifferentiation to an alternative differentiated cell type, leading to the emergence of new phenotypes [13,14,15,16,17]. Of late, it has been proposed that the disruption of mitochondrial physiology is a novel mechanism, which, under specific cellular conditions, leads to lineage plasticity and may be one way that tumor cells establish resistant phenotypes to therapeutic interventions [18,19]. Therefore, we ask, does lineage plasticity, due to altered mitochondrial detoxification enzymes such as MnSOD, also lead to a therapy resistance phenotype in a subgroup of ER+ LuBCa IDCs? Furthermore, under specific circumstances when MnSOD is dysregulated or deleted, does it contribute to aberrant cellular metabolism and/or mitochondrial bioenergetics, oncogenesis, and/or therapy resistance?

## 2. Post-Translational Deacetylation of MnSOD by SIRT3

Sirtuins are NAD^+^ dependent class III histone deacetylases that are found in various genomes from bacteria to humans [20,21,22], which deacetylate a wide range of downstream non-histone targets, including transcription factors, metabolic enzymes, and detoxification enzymes. In addition, several sirtuins have also been shown to deacetylate histones under specific conditions, including SIRT1, SIRT2, and SIRT6 [23,24,25,26]. Sirtuin proteins play important roles in metabolic and bioenergetic processes, stress resistance, cell survival, and aging [27,28]. Sirtuin genes are human and murine homologs of the *Saccharomyces* cerevisiae *Sir2* that have been shown to regulate life span and aging in model organisms, including *Saccharomyces cerevisiae*, *C. elegans,* and *Drosophila melanogaster* [23,29,30,31]. The sirtuin family, which constitutes the main mammalian lysine acetyl transferases, consists of seven NAD^+^-dependent enzymes. These seven proteins are localized to the nucleus (*Sirt1*, *6,* and *7*), mitochondria (*Sirt3*, *4,* and *5*), and cytoplasm (*Sirt2*) and share a 275-amino-acid catalytic deacetylase domain [27].

Sirtuins, including the primary mitochondrial sirtuin, Sirt3 [21,22], appear to respond to changes in cellular and nutrient conditions, including mitochondrial stress, resulting in the activation of their deacetylase or ribosyltransferase activity, and consequently, in a post-translational modification (PTM) of downstream target proteins acetylation (Ac) levels [20,32,33,34,35]. Lysine acetylation neutralizes the positive charge on the lysine residue and can cause structural and functional changes in the protein due to changes in electrostatic potential. We [36], and others [34,37,38], have shown that MnSOD is an important downstream target of SIRT3 in its role and as a mitochondrial fidelity protein [21,33,39,40]. A key aspect of sirtuins is that they require NAD+ as a co-factor, which makes them metabolic sensors and connects their enzymatic activity to the energy and redox state of cells [41,42,43]. Most mitochondrial proteins are autoacetylated due to a high acetyl-CoA concentration in mitochondria, and protein functions modified by lysine acetylation are mainly regulated by the activity of deacetylation enzymes such as SIRT3 [36,44,45,46].

Thus, an important theme that has emerged in the last several years is that SIRT3, and its most important downstream targets, direct multiple mitochondrial processes. In this regard, it appears that the non-physiological levels of MnSOD acetylation (Ac), at least in part, connect metabolic and bioenergetic balance and tumor cell growth and survival. This is based on a recently identified novel mitochondrial signaling axis centered on MnSOD-Ac, which, when dysregulated, disrupts cell metabolism, leading to aberrant ROS levels [47,48]. MnSOD is a mitochondrial matrix-localized homotetrameric antioxidant enzyme with four identical subunits, each harboring a Mn^2+^ atom [49,50]. The primary function of MnSOD is to scavenge superoxide generated from metabolic processes, including the electron transport chain. Mammalian MnSOD appears to have four lysines that have been identified as potential SIRT3 downstream targets, including K53, K89 [34,51], K68 [38,52,53], and K122 [34,35,37,38], using different methods, including site directed acetylation mutants, physical lysine acetylation followed by mass spectrometry analyses, and acetyl-lysine specific monoclonal antibodies. However, the biochemical and physiological significance of each of these lysines, and the molecular mechanism directing MnSOD enzymatic activity and mitochondrial metabolism, remains to be fully determined. The SIRT3-MnSOD-Ac axis is an active area of research in the regulation of mammalian and human cells/organs and its dysregulation appears in several human illnesses, including cancer. It now appears quite clear that the SIRT3-MnSOD-Ac axis is a mitochondrial signaling hub that regulates how cells adapt to ROS-induced metabolic stress in addition to reprograming mitochondrial metabolism, which may play an important role in late-onset diseases [44,54,55,56,57]. However, there are limited data to demonstrate the mechanism behind this idea [36,45].

Recently, we have been focusing on MnSOD-K68-Ac as one of the key SIRT3 targets that may play an important role in the oncogenicity observed in cells lacking SIRT3. In this regard, mice lacking *Sirt3*, and thus containing MnSOD-Ac, develop estrogen-receptor-positive (ER+), poorly differentiated, high Ki-67 mammary gland tumors that exhibit very similar characteristics to human luminal B breast malignancies [33,36,44,56]. As compared to luminal A ER+ breast cancers, luminal B subtypes tend to have increased proliferation markers and, most importantly, can exhibit a tumor cell endocrine-resistant phenotype [45]. In this regard, there are nearly thirty publications that suggest a role for the SIRT3-MnSOD-Ac signaling axis in the dysregulation of mitochondrial function and dysregulation. Interestingly, mice that have a monoallelic knockout for MnSOD (MnSOD^+/−^) exhibit decreased MnSOD activity, increased oxidative stress, decreased life span, as well as aging-related phenotypes, especially carcinogenesis, which includes breast malignancies [58].

## 3. Lysine 68 Acetylation-Dependent Regulation of MnSOD Enzymatic Activity

The mitochondria electron transport chain (ETC) directs the flow of electrons in normal cellular metabolism, resulting in the production of superoxide radicals at less than 1% of the total rate of electron transport from NADH to oxygen [59,60,61]. A high level of superoxide can damage many components of cells, such as proteins, DNA, RNA, and lipids [62,63,64] potentially leading to cell death or dysfunction which can result in human illness permissive phenotypes. In addition, maintaining the low steady-state level of superoxide is critical for proper regulation of mitochondria function [65]. Therefore, the peroxide-removing system in mitochondria is required for respiration and to maintain metabolic homeostasis [62]. MnSOD is one of the critical mitochondrial detoxification enzymes neutralizing the toxic superoxide to less harmful and membrane-permeable hydrogen peroxide, which is converted to oxygen and water by peroxidases, such as catalase, in the cytoplasm. Two molecules of O_2_^•−^ are oxidized to oxygen O_2_ and H_2_O_2_ through the catalytic reduction of Mn^3+^ to Mn^2+^ and restoration of Mn^2+^, respectively [61,66,67]. Due to the inducible oxidative stress of O_2_^•−^ produced during energy production in mitochondria, MnSOD detoxification/ROS scavenging enzymic activity is critical to ATP production, maintaining mitochondrial homeostasis, and cell survival.

In order to regulate the activity of proteins, cellular signaling networks employ different mechanisms, including post-translational modifications (PTMs), such as acetylation [68,69,70,71]. Lysine acetylation is an extensively used PTM to regulate transcription and signaling factors, as well as histones regulated by specific sirtuins [38,72]. Non-acetylated lysine moieties are positively charged [66], while acetylation of these lysine residues, through a PTM, neutralizes this charge and alters the enzymatic function [67,73]. In general terms, the acetylome is roughly 60% that of the kinome and studies have identified the presence of more than 2000 acetylation substrates localized in both the cytosol and mitochondria, indicating the critical role of acetylation in cellular regulation [38,74,75,76,77]. In this regard, acetylation is one of the most frequent PTMs in the mitochondria.

We recently published data, using site-directed MnSOD mutants, where K68 was substituted with either a glutamine (Q) [78,79], an acetyl-lysine mimic mutant, or an arginine (R), a deacetylated lysine mimic mutant [80]. MnSOD^−/−^ MEFs infected with lenti-MnSOD^K68Q^ decreased activity, while infection with lenti-MnSOD^K68R^ showed a significant increase in MnSOD activity, as compared to MEFs infected with wild-type (WT) lenti-MnSOD^WT^ [34,35,38]. In addition to these results, further experiments with our anti-MnSOD-K68-Ac antibody showed that (1) caloric restriction, (2) time-restricted fasting (36 h) [35], and (3) forced exercise [80] deacetylated K68, identifying K68-Ac as a physiologically relevant post-translational modification. These results led us to explore both the mechanism underlying how SIRT3 regulates acetylation of lysine-68 and directs MnSOD activity, as well as its role in oncogenicity and tumor cell resistance. Using the known crystal structure of MnSOD (PDB: 2adp), we conducted computational structural biology studies to examine both MnSOD^K68Q^ acetylation mimic mutant, as well as MnSOD-K68-Ac, to determine any differences in charge distribution, compared to the MnSOD wild-type.

We have previously published that *MnSOD^K68Q^* and MnSOD-K68-Ac have nearly identical physiological and biochemical properties using tissue culture, molecular biology, and codon substitution experiments in bacteria [52]. In addition, we have also used the molecular dynamics core to calculate the surface charge density for MnSOD^WT^ and MnSOD-K68-Ac, which clearly showed that K68-Ac increases the negative surface charge distribution along the α1/α2 helices adjacent to the Mn^2+^ binding site/tetramerization interface of MnSOD-K68-Ac [52]. Importantly, an identical change in surface charge was also observed for MnSOD-K68-Ac and MnSOD-K68A by others, including Lu et al., 2017 (see Figure 2) [81] and Lu et al., 2015 (see Figure 7 [49]. The tetrameric interface is a four-helix bundle, symmetrically composed of two sets of helices (α1 and α2) separated by a tight turn. These calculations showed that these alpha helices, α1 and α2, which already have comparatively low surface charge, see a further decrease in surface charge upon acetylation of K68. Given that these helices make up the majority of the tetramer interface, the decreased surface charge may lead to destabilization of the quaternary structure. Thus, these results indicate that the MnSOD-K68Q mutant mimics the molecular dynamics of K68-Ac and suggest how acetylation of K68 might disrupt MnSOD detoxification activity by disrupting the organization of the homotetrameric complex. Further computational studies focusing specifically on the stability of the tetrameric interface of the MnSOD complex with respect to the acetylation status of K68 are required to conclusively demonstrate this proposed mechanism. The immediate result of the breakdown of the tetrameric structure is likely the formation of two dimeric species. However, there is evidence to suggest that the dimer is less stable and the equilibrium between the dimeric and monomeric species for eukaryotic MnSOD is rapid [82]. For consistency, we will refer to the degraded tetramer as “monomer”, even though it could exist in either monomeric or dimeric form.

Experiments with the MnSOD-K68-Ac mimic mutant, as well as the deacetylation mimic mutant, have indicated that MnSOD-K68Q loosens the homotetramer and dimeric/monomeric forms are favored. Based on these results it was surmised that MnSOD-K68-Ac may have a different function than the detoxification SOD activity observed for the homotetrameric complex. In this regard, the Flag-tagged *MnSOD* constructs (*MnSOD^WT^*, *MnSOD^K68R^*, and *MnSOD^K68Q^*) were immunoprecipitated (IPed) and tested for enzymatic activity. These experiments showed that MnSOD from MEFs lacking *MnSOD* (MnSOD^−/−^) expressing *MnSOD^K68Q^* exhibited a 40-fold increase in peroxidase activity, compared to IPed MnSOD-K68R or MnSOD-WT [52]. Identical results were achieved in codon substitution experiments using MnSOD-WT and MnSOD-K68-Ac recombinant proteins. Size exclusion chromatography was used to separate the tetramer (96 kDa) and monomer forms (24 kDa) and subsequent immunoblotting showed that the tetramer was enriched in MnSOD-WT, while MnSOD-K68-Ac is mostly in a monomeric form. The separate fractions were then analyzed for dismutase and peroxidase activity and overwhelmingly the tetramer showed dismutase activity, while the monomer showed peroxidase activity. Based on these results, it appears that MnSOD exhibits a dichotomous function, based on its lysine 68 (K68) acetylation status, where the deacetylated homotetrameric form acts as a protective detoxification enzyme against persistent/aberrant ROS. In contrast, K68-Ac inhibits homotetramer formation and, therefore, shifts the MnSOD equilibrium towards a predominantly monomeric form that functions as a peroxidase. As such, the working hypothesis is that K68-Ac not only inhibits its detoxification activity, but also results in a switch in enzymatic function [52,53].

## 4. MnSOD^K68Q^ Is a Monomer That Exhibits Oncogenic Properties

We previously showed that mice lacking *Sirt3* develop ER+ luminal B-like tumor properties, suggesting that MnSOD, perhaps when acetylated, may function as a tumor promoter, instead of its more traditional function as a detoxification enzyme and tumor suppressor (TS) [83,84]. For initial experiments, an in vitro model was used, in which at least two oncogenes, i.e., *c-Myc* or *Ras* [85], are required to immortalize and/or transform primary rodent cells. MnSOD^−/−^ primary mouse embryo fibroblasts (pMEFs) infected with lenti-MnSOD^K68Q^, and *c-Myc* or *Ras*, became immortalized (i.e., divided beyond 15 cell passages) but not pMEFs infected with *c-Myc* or *Ras* alone. In contrast, co-infection of lenti-MnSOD^K68R^ with *c-Myc* or *Ras* failed to immortalize pMEFs [35,52]. As a positive control, pMEFs infected with both *c-Myc* and *Ras* were immortalized, as has been previously shown [85,86]. Interestingly, infection with lenti-MnSOD^K68R^ prevented immortalization of pMEFs with *c-Myc* and *Ras*, implying that MnSOD^K68R^ is a tumor suppressor. Lastly, pMEFs infected with lenti-MnSOD^K68Q^, as compared to cells infected with MnSOD^K68R^, exhibited increased: (1) soft agar growth, a measure of anchorage independence; (2) growth when plated at low density (250 cells/60 mm plate), a measure of proliferative capacity; (3) doubling time, a measure of proliferative rate; and (4) xenograft growth, a measure of tumorigenicity [52,53].

These established in vitro tissue culture transformation experiments support the premise that MnSOD-K68-Ac can function, under specific conditions, as a tumor promoter [52,87]. Thus, these data strongly imply that *MnSOD^K68Q^*, which is proposed to model MnSOD-K68-Ac, can function as an in vitro oncogene as opposed to its more established role as a detoxification enzyme that can, under specific conditions, function as a tumor suppressor. These results may explain why initial clinical data suggest that MnSOD acts as a tumor suppressor [88]; however, surprisingly, there are also relatively new convincing data that also show that high MnSOD levels positively correlate with more aggressive tumors [89,90]. These studies, and our data, suggest a dichotomous role for MnSOD, where the tetramer acts as a TS during the early, proliferative stage of tumor initiation. However, once oncogenesis progresses, monomeric K68-acetylated MnSOD establishes a tumorigenic phenotype.

**Figure 1:**
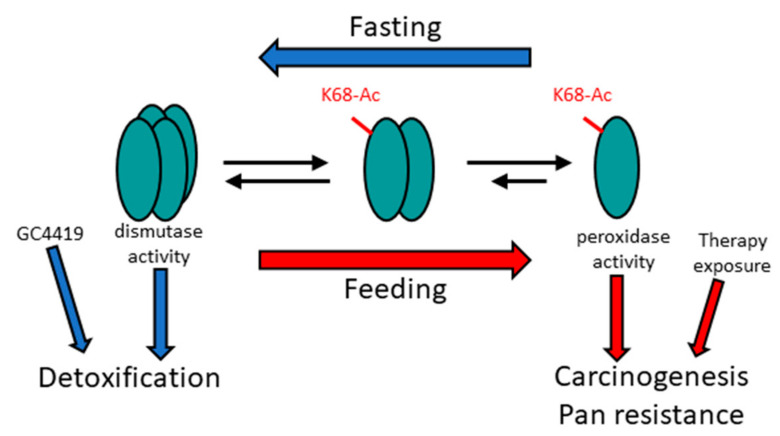
**Proposed mechanism of MnSOD detoxification and peroxidase roles.** The tetrameric detoxification complex plays a role in a fasting physiology (blue arrows), whereas increased monomeric MnSOD, and its peroxidase activity, may be required for mitochondrial reprogramming to generate energy in a feeding state (red arrows). Other genetic and/or other types of cell stress may also create an oncogenic phenotype, which reprograms the mitochondria by increasing MnSOD-K68-Ac.

Thus, MnSOD may switch from a tetrameric dismutase to a monomeric peroxidase in response to specific cellular stress conditions, such as nutrient status and/or transformation (Figure 1). In this model, we posit that genetic or metabolic abnormalities dysregulate MnSOD-Ac and shift the balance towards higher monomer levels with decreased tetramer. The MnSOD-Ac, while accommodating the metabolic needs of the cell, may also function as a tumor promoter under specific conditions. Lastly, it is surmised that the SIRT3-MnSOD signaling axis may also be a potential therapeutic molecular target, including agents such as GC4419 (Galera Therapeutics, Inc. Malvern, PA, USA), which chemically dismutase/detoxify superoxide instead of enzymatic removal by MnSOD [87].

Considering the important role of MnSOD in detoxification, it is worthwhile to mention that the role of MnSOD goes beyond this activity. Van Remmen et al. revealed that MnSOD^+/−^ heterozygous mice had a 50% reduction in SOD activity and increased 8-oxo-2-deoxyguanosine (8oxodG) in nuclear DNA [54,55,58], suggesting the enhancement in oxidative stress in monoallelic MnSOD^+/−^ tissues. Tumor formation was observed in all MnSOD^+/−^ mice with increased oxidative DNA damage, which indicates the correlation of MnSOD activity and carcinogenesis [59,91]. Interestingly, *Sirt3^−/−^* MEFs also showed an elevation of oxidative stress induction, which is associated with abnormal physiology changes in mitochondria. Additionally, ER/PR-positive mammary tumor development was observed in mice lacking *Sirt3* (34). Other studies also showed an increase in Ki-67 in knockout *Sirt3* female mice ER+ tumors, a marker for human luminal B breast malignancies [33,35,45]. As previously mentioned, we proposed the equilibrium shift model from MnSOD tetrameric form to monomeric form when K68 is acetylated [52,87]. Further results revealed that MnSOD-K68-Ac functions as a tumor promoter in cells expressing MnSOD^K68Q^, the MnSOD acetylation mimic mutant. Together, these results show a correlation of *Sirt3*, MnSOD acetylation status, and ROS detoxification activity, as well as mitochondrial metabolic stress with carcinogenesis, implicating *Sirt3* as a tumor suppressor.

## 5. Redox Signaling Involving MnSOD

As discussed above, it is proposed that there may be a mitochondrial equilibrium shift in the MnSOD tetrameric/monomeric ratio when K68 is acetylated. MnSOD catalyzes the dismutation of superoxide (O_2_^•−^) into hydrogen peroxide (H_2_O_2_); however, it has been shown to demonstrate peroxidase activity under certain conditions [52,92,93]. MnSOD is highly regulated at the transcription level by NF-kB, SP1, AP1, AP2, cytokines, and protein kinase C and is strongly correlated with cell cycle [50,94]. MnSOD is also post-translationally regulated by acetylation and phosphorylation [62,94]. Given the reversibility of the active site, tight regulation of expression and activity, and the added fact that the electron transport chain is riddled with antioxidants and only a small production of O_2_^•−^, it has been suggested that MnSOD’s second important function is as a signaling hub, as well as a superoxide dismutase [95]. This is supported by peroxidase activity [52] and the specific and selective nature in which O_2_^•−^ and H_2_O_2_ oxidize targets [96,97]. H_2_O_2_ has a long half-life in aqueous solutions and can easily permeate mitochondria membranes; however, due to varied reduction potentials of amino acids, it has been shown that H_2_O_2_ selectively binds and oxidizes the active site of several phosphatases, rendering them inactive [96].

Increased MnSOD activity also leads to increased H_2_O_2_ signaling, inactivation of phosphatases under specific cellular and mitochondrial conditions, and an increase in phosphorylation activity of JNK/c-Jun and AMPK [96]. Overexpression of MnSOD in Jurkat cell lines led to increased phosphorylation of JNK and downstream c-Jun [96]. This pathway upregulates the expression of inflammatory cytokines and acts as a positive feedback loop for MnSOD as c-Jun is an AP-1 subunit that upregulates expression of MnSOD. JNK has been shown to prevent tumor initiation [98] and, through the activation of this pathway, MnSOD acts as a tumor suppressor. In addition, overexpression of MnSOD, confirmed with exogenous addition of H_2_O_2_, led to oxidation of CaMKII-M281/282, consequently activating CaMKII [90]. This leads to phosphorylation and activation of AMPK, subsequent phosphorylation, and inactivation of acetyl-CoA carboxylase (ACC), and an increase of glycolysis [90,99]. The switch to glycolysis, i.e. the Warburg effect, is a hallmark of cancer metabolism [100,101] and, in this instance, MnSOD is acting as a tumor promoter, specifically in breast cancer, as increased MnSOD expression and glycolysis both increased as severity of breast malignancies [90]. Short-term calorie restriction can also increase H_2_O_2_ levels, activating AMPK [98]. The combination of results from Han, D. et al. 2020 and Han, L. et al. 2020 [102,103] show a positive feedback loop with increased SIRT3 leading to activation of the AMPK pathway and phosphorylated AMPK upregulating SIRT3 expression. [103] Phloretin was shown to mitigate the oxidative stress induced by palmitic acid and increase phosphorylation of AMPK [103]. This also correlated to increased expression of SIRT3, which was reduced when an AMPK inhibitor (dorsomorphin) was used [103].

Alternatively, inactivation of MnSOD leads to a buildup of O_2_^•−^, which can also act as a signaling molecule that, under specific conditions, can oxidize and inactivate PTEN [104,105]. The buildup of O_2_^•−^ due to the inactivation of SODs combines with nitric oxide to form peroxynitrite and nitrosylate PTEN, rendering it inactive [104,105,106]. This leads to increased phosphorylated (active) AKT to support cell growth. As a negative feedback loop, peroxynitrite can directly oxidize MnSOD-Tyr34, inactivating the enzyme [107]. In ER+ breast cancer, it has been reported that estrogen signaling relocates ER to the mitochondria, where it binds to MnSOD [105]. This blocks SIRT3 from binding and/or deacetylating MnSOD. The O_2_^•−^ build-up leads to phosphorylation and activation of mTORC2, which phosphorylates and activates downstream targets AKT and PKC-α [105]. Hyper-acetylation of MnSOD can lead to over activation of AKT. Because AKT is often overactive in cancers, increasing cell survival and proliferation [108], active deacetylated MnSOD would be acting as a tumor suppressor. The totality of these results demonstrates the complexity of MnSOD’s roles in cell growth and strongly implies that PTMs can significantly determine the reparative or damaging effects of this detoxification enzyme.

## 6. MnSOD Signaling Linked to Lineage Plasticity

MnSOD has also been linked to signaling in cancer cells through the epithelial to mesenchymal transition (EMT), also referred to as a lineage-plasticity-like phenotypic switch, leading to more aggressive phenotypes, pan anticancer resistance properties, and a metastasis permissive tumor cell phenotype [109,110,111]. There is a positive correlation in MnSOD expression and EMT score of all subtypes of breast cancer and the subsequent knockdown of MnSOD in mesenchymal cells decreases their EMT markers and morphology [111]. Conversely, it was shown that FeTPPS (a peroxynitrite scavenger) mitigates the MET (mesenchymal–epithelial transition), the reversal of EMT, and the effect of *siMnSOD*. Similarly, in small-cell lung cancer (SCLC)-derived cancer stem-like cells, overexpression of MnSOD and FOXM1 mitigated the effects of genistein, decreasing stemness/lineage-plasticity-like markers and the profound migratory/invasive activity [109], whereas knockdown of *MnSOD* and *FOXM1* enhanced the effect of genistein [109]. These studies all support the hypothesis that an increase in MnSOD can potentially lead to an increase in mitochondrial H_2_O_2_ concentrations, acting as a signaling molecule that increases expression of stemness/lineage-plasticity-like markers; however, the actual MnSOD activity in cells is not addressed in these experiments.

In a quite interesting study, it was shown that the forced overexpression of MnSOD also led to an increase in acetylation of lysine 68 in breast cancer cells [53]. He et al. showed that the acetylation of MnSOD correlates with the upregulation of stemness/lineage plasticity-like biomarkers Oct4, Sox2, and Nanog through HIF2α signaling. However, He et al. also showed that the inhibition of H_2_O_2_ signaling with overexpression of catalase reduces the expression of HIF2α, as well as stemness biomarkers, including Oct4 and Nanog [53]. Based on these results, they postulated that MnSOD-K68-Ac leads to higher H_2_O_2_ levels, which adds intriguing possibilities when compared to previous reports [83,112,113]. Interestingly, the *MnSOD^K68Q^* mutant, which is a biochemically validated acetylation mimic, also showed an increase in stemness markers [53] and provided more supporting data regarding the increased MnSOD acetylation hypothesis. A study in squamous carcinoma cells showed that the invasive cell line (A431-III) had inherently more expression of MnSOD and higher H_2_O_2_ levels than the parental cell line (A431-P) [110]. Using siRNA, the knockdown of *MnSOD* led to an increase in H_2_O_2_ and invasiveness of the cells in both the parental and metastatic cell line [110]. Treatment with antioxidants, diphenyleneiodonium, and N-acetyl-l-cysteine blocked H_2_O_2_ signaling and reversed the effects of the siRNA [110]. These two papers showed that either the acetylation or the downregulation of MnSOD leads to higher mitochondrial H_2_O_2_ concentrations, which in turn leads to a potential lineage-plasticity-like switch to cells that exhibit stemness or EMT biomarkers.

Shedding light on the seemingly opposing nature of MnSOD, as it was previously discussed when cells are manipulated to express MnSOD-K68-Ac experimentally by using the acetylation mimic *MnSOD^K68Q^* or made in bacteria using a codon expansion method, the concentration of monomeric MnSOD also increased as a proportion and/or change in the tetrameric to monomeric ratio [52]. In addition, the monomer form of MnSOD shows peroxidase activity instead of the more established superoxide dismutase activity [52,92]. Instead of acetylation being an on/off switch, the working hypothesis is that acetylation can cause a biological change of function. This hypothesis supports the theory of MnSOD being a signaling hub. Expressing the *MnSOD^K68Q^* acetylation mimic mutant or silencing *SIRT3* also shows a higher proportion of monomer and peroxidase activity [52]. A potential extension of these results could be that when MnSOD is overexpressed beyond its physiologically normal levels, a disproportionate increase in monomeric MnSOD occurs, which functions as a peroxidase [52]. Therefore, when it is silenced, as in Fan et al. [110], H_2_O_2_ concentration increases. The conditional nature of MnSOD all falls back to whether it is acetylated or not, and on the functionality of SIRT3.

## 7. The Cofactor of MnSOD and Peroxidase Activity, Another Layer of Regulation

MnSOD is an old, evolutionally conserved protein and is required for organisms that make ATP through respiration, primarily coming in two varieties that are categorized based on the cofactor identity. Eukaryotic MnSOD utilizes a Mn^2+^ cofactor, whereas bacteria can utilize both MnSOD and a distinct but similar FeSOD using Fe^3+^ as a cofactor. The essential structure of these enzymes is remarkably similar, especially in the active site region. In both, the active site region contains the metal cofactor ligated by three histidine residues, one aspartate residue, and one hydroxide ion that is stabilized by a highly conserved glutamine residue. In MnSOD, the glutamine arises from the C-terminal domain (Q146 *E. coli*), but in FeSOD, the glutamine arises from the N-terminal domain (Q69 *E. coli*) [114]. Despite the similarity of the enzyme structures, the proteins behave very differently with respect to incorporation of the metal cofactor.

It has been well established that substitution of iron into MnSOD, (Fe(Mn)SOD), leads to a virtually complete loss of dismutase activity for the enzyme. It is also well understood that the structure of the enzyme is virtually unchanged upon incorporation of the “wrong” metal and the loss of activity most likely arises from a deficiency in the reduction potential of the Fe-substituted enzyme. Vance et al. suggested that Fe^2+^(Mn)SOD is able to catalyze the reduction of O_2_^•−^, but the Fe^3+^(Mn)SOD generated from Fe^2+^(Mn)SOD during the previous reaction is not able to oxidize O_2_^•−^, and the catalytic cycle cannot be completed [115]. A similar argument can be made for Mn(Fe)SOD, which is also inactive as a dismutase. Thus, metalation is very important to these enzymes and various studies have been conducted to determine the consequences of mis-metalation. The specificity of MnSOD for the Mn cofactor is quite low. In fact, in bacteria, Fe(Mn)SOD is in 10–100 times greater abundance than MnSOD and the ratio of MnSOD/Fe(Mn)SOD is only increased under conditions of oxidative stress, such as the time when the catalytically active enzyme is needed [116]. However, within mitochondria, the enzyme is almost exclusively bound to the Mn^2+^ cofactor under normal conditions. Studies with *S. cerevisiae* have suggested that the exclusivity of MnSOD within mitochondria arises because the metalation process is coupled with the import of the unfolded polypeptide MnSOD into the mitochondria [116], and once the cofactor is incorporated into the enzyme, its removal has a high energy barrier.

Mis-metalation can be induced in *S. cerevisiae* by disruption of manganese trafficking factor (mtm1) [117]. Mtm1 plays a role in the insertion of Mn into the MnSOD enzyme and deletion of mtm1 has been shown in vivo to increase the concentration of Fe(Mn)SOD without reducing the concentration of mitochondrial manganese. Disruption of mitochondrial iron homeostasis, specifically via knockdown of *ssq1* or *grx5* [118], has also been shown to affect the MnSOD/Fe(Mn)SOD ratio in cells. Mis-metalation of MnSOD is also observed under conditions of Mn scarcity as the Fe-incorporated inactive enzyme is preferred over the apo-enzyme [119]. This is especially the case in cells where MnSOD is overexpressed [93,115,118].

Recent developments suggest that Fe(Mn)SOD has deleterious effects, not only because of its loss of dismutase function, but also because of a gained pro-oxidant/peroxidase function [120]. In early studies, this peroxidase function was attributed simply to overexpressed MnSOD, based on the oxidation of Amplex Red when MnSOD is incubated with H_2_O_2_ [92]. However, that interpretation has drawn some criticism [121] and several reports have shown that MnSOD is unable to oxidize ABTS^∙+^ [120] or Amplex Red [122], whereas Fe(Mn)SOD is able to oxidize those reagents. Ganini et al. showed that, in bacteria, the peroxidase activity is highly correlated to the concentration of Fe(Mn)SOD and the dismutase activity is highly correlated to the concentration of MnSOD [93]. Further, Ganini explored the metalation of MnSOD in mammalian cells cultivated in media with different iron to manganese ratios [122]. Cells grown in Mn-deficient media were observed to have significant iron incorporation, whereas cells grown in Mn-supplemented media were mainly bound to Mn. The cultivated Fe(Mn)SOD was then shown to catalyze the oxidation of Amplex Red in the presence of H_2_O_2_ and supported protein radical formation. The cultivated Mn-bound MnSOD was unable to oxidize Amplex Red and the formation of protein radicals was not observed [122]. Thus, the current evidence suggests that Fe incorporation into the MnSOD enzyme is a critical factor in the observed peroxidase activity and the role of overexpression of MnSOD is simply that it leads to a Mn deficiency in cells and makes mis-metalation more likely.

## 8. Conclusions

**Figure 2:**
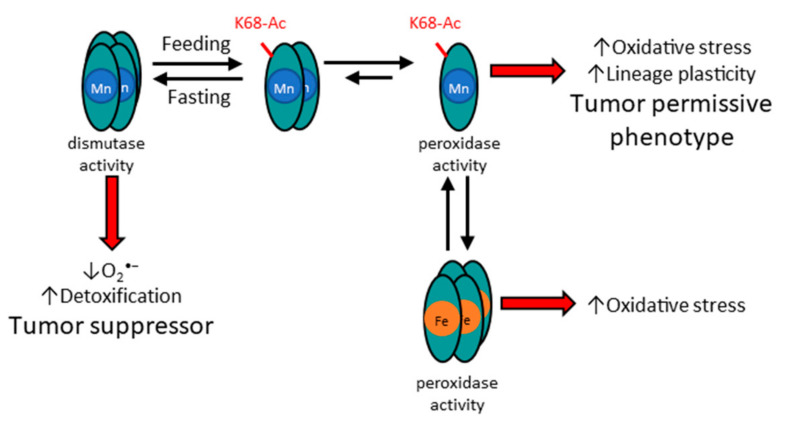
**Acetylation status of SOD2 affects structure and function, as well as metalation status**. The potential mechanism for how acetylation of MnSOD lysine 68 alters the structure and function of MnSOD enzymatic activity. In this regard, it is proposed that acetylation, for example through feeding, leads to the formation of a dimer. In addition, it is surmised that mitochondrial damage via oxidative stress, or other forms of stress, may lead to iron incorporation of MnSOD.

In conclusion, the data in this review article suggest that MnSOD-Ac, including K68-Ac, may function as an oncoprotein in an advanced stage of cancer, in contrast to the more traditional premise that MnSOD is a detoxification TS (Figure 2), and this dual function may arise from the presence of two structural forms of MnSOD. As such, it is surmised that acetylation status of MnSOD-K68 is a molecular switch that directs its enzymatic detoxification from its antioxidant activity (i.e., metabolic stress/protective function) as a tumor suppressor (tetrameric) or as a oncoprotein (monomeric) and, most importantly, that may promote a tumor-permissive phenotype through lineage-plasticity-like properties. This idea has, over the last decade, led to multiple publications suggesting a potential link between mitochondrial function, and dysregulation, in the lineage cell fate and plasticity in both stem cells, as well as tumor cells. It also expands the theme that the mitochondria, perhaps through small reactive oxygen species (ROS), may relay signals to the other parts of the cell to maintain both metabolic and bioenergetic equilibrium. The disruption of these pathways, through one of many potential processes, may lead to permissive phenotypes for human illness, including malignancies.
